# Detection and Mosaicing Techniques for Low-Quality Retinal Videos

**DOI:** 10.3390/s22052059

**Published:** 2022-03-07

**Authors:** José Camara, Bruno Silva, António Gouveia, Ivan Miguel Pires, Paulo Coelho, António Cunha

**Affiliations:** 1 Departamento de Ciências e Tecnologia, University Aberta, 1250-100 Lisboa, Portugal; 1701367@estudante.uab.pt; 2 Institute for Systems and Computer Engineering, Technology and Science (INESC TEC), 4200-465 Porto, Portugal; acunha@utad.pt; 3 Polytechnic of Leiria, 2411-901 Leiria, Portugal; 2182734@my.ipleiria.pt; 4 Escola de Ciências e Tecnologias, University of Trás-os-Montes e Alto Douro, Quinta de Prados, 5001-801 Vila Real, Portugal; jgouveia@utad.pt (A.G.); impires@it.ubi.pt (I.M.P.); 5 Instituto de Telecomunicações, Universidade da Beira Interior, 6200-001 Covilhã, Portugal; 6 Institute for Systems Engineering and Computers at Coimbra (INESC Coimbra), DEEC, Pólo II, 3030-290 Coimbra, Portugal

**Keywords:** convolutional neural networks, retinal screening, fundus imaging, object detection, mosaicing

## Abstract

Ideally, to carry out screening for eye diseases, it is expected to use specialized medical equipment to capture retinal fundus images. However, since this kind of equipment is generally expensive and has low portability, and with the development of technology and the emergence of smartphones, new portable and cheaper screening options have emerged, one of them being the D-Eye device. When compared to specialized equipment, this equipment and other similar devices associated with a smartphone present lower quality and less field-of-view in the retinal video captured, yet with sufficient quality to perform a medical pre-screening. Individuals can be referred for specialized screening to obtain a medical diagnosis if necessary. Two methods were proposed to extract the relevant regions from these lower-quality videos (the retinal zone). The first one is based on classical image processing approaches such as thresholds and Hough Circle transform. The other performs the extraction of the retinal location by applying a neural network, which is one of the methods reported in the literature with good performance for object detection, the YOLO v4, which was demonstrated to be the preferred method to apply. A mosaicing technique was implemented from the relevant retina regions to obtain a more informative single image with a higher field of view. It was divided into two stages: the GLAMpoints neural network was applied to extract relevant points in the first stage. Some homography transformations are carried out to have in the same referential the overlap of common regions of the images. In the second stage, a smoothing process was performed in the transition between images.

## 1. Introduction

Many eye-related diseases are degenerative. They progressively aggravate the patient’s clinical situation and are irreversible, leading to visual impairments or blindness. There is the need for early action, which prevents, minimizes, or leads to the search for specialized screening and medical diagnosis. Fundus cameras are professional equipment used to capture the eye’s retina and diagnose eye-related diseases. Such professional equipment produces high-quality fundus images, and due to such quality, this equipment’s usage is widespread for medical use.

From another perspective, the lack of means for healthcare or equipment in areas with limited economic resources leads to the increasing trend of severity in health-related problems in such populations. Low-cost alternatives have been developed in the last few years to mitigate this problem. Some alternatives might be the D-Eye [1], Peek Retina [2] and iNview [3]. In particular, the D-Eye device is magnetically coupled to a smartphone and can capture retinal videos with sufficient quality to perform a pre-screening. It is a low-cost lens that can be attached to the lens of a smartphone to get undisturbed pupil background photos and videos with the added advantage of bringing more comfort to the patient. However, it does not have the necessary sharpness when used in eyes with small pupils in eyes with the opacity of media (keratitis, cataract). Nevertheless, against the equipment’s reduced price and portability advantages, it presents the drawback of a smaller field-of-view and lowers resolution images compared to professional fundus machines.

From the resulting data, it is possible to observe the further need to perform a professional and more accurate screening process, keeping in mind that the earlier the medical diagnosis is performed, the lesser the disease impact will be. An opinion/preference study, made to medical students, revealed 92% use preference of D-Eye lens compared with a direct ophthalmoscope [4].

**Figure 1:**
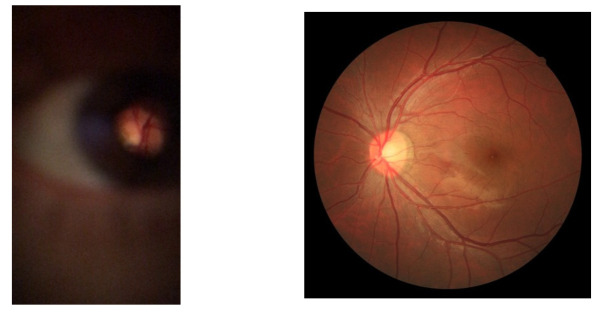
Image acquired with D-Eye lens (**left**) and image of FIRE public dataset [5] (**right**).

An example of a D-Eye captured image is presented in Figure 1 at the left and can be compared to a professional fundus equipment image (at the right). It is possible to observe in the figure at left that only a tiny portion of information (retinal area) is essential for the medical diagnosis. Also, the remaining parts of the image might be disposable compared to the image in the right, where there’s a broader informative area with better image quality. The last picture was gathered from the FIRE public dataset [5], and it presents an example of a capture from a professional fundus device.

As contributions, this paper presents a pipeline targeting two tasks:

- The first task is a framework focused on the detection of lower-resolution retinal images taken with a smartphone equipped with a D-Eye lens. Here, two methods were proposed and compared: a classical image processing approach and a YOLO v4 neural network. To explore this task, a private dataset presented by Zengin et al. [6], which contains 26 retina videos around the optic disc, with lower-resolution images, and annotated two subsets, one with the localization of the visible retinal area and other with vessel segmentation was used;
- The second task explored in this paper is the mosaicing technique in images captured from devices attached with D-Eye lenses so that a summary image can be provided as the result of some retinal video. It was explored the Glampoints model proposed by Truong et al. [7] applied to the images resulting from the previously described task.

The document is organized as follows: Section 2 presents a brief description of some of the related work references, Section 3 presents the material and methods of the proposed pipeline, including the datasets, data preparation for the explored methods, including training and evaluation of the neural networks. Next, in Section 4 are presented the results obtained from the proposed methods. Finally, in Section 5, conclusions of the developed work are presented.

## 2. Related Work

In this section, a brief presentation of relevant references for this work is presented, separated in both Object Detection and Mosaicing algorithms.

### 2.1. Object Detection

The main goal of this technique is to determine from a set of classes which ones are present in the input image or video and their location [8]. Furthermore, this task can provide individuality to each object detected instead of assigning it to a class. For every detected object, the algorithm will provide a bounding box that will surround the object and identify it with a label of the target class name. These can be divided into two-stage detectors and one-stage detectors.

The two-stage detectors are object detectors divided into two individual phases, including the proposal region and the classification. The first step will propose areas of the input image where it is more probable to contain an object. In the second step, the features from the selected regions by the proposal region step are extracted to assign a classification to the found objects. Unlike the previously presented, the one-stage detectors output results in a single stage, i.e., these do not have the region proposal algorithm step. Due to the fewer stages of this method, this kind of detector usually achieves higher detection speeds when compared to two-stage detectors [9]. Still, it has less accuracy detecting smaller-sized objects [8].

**Figure 2:**
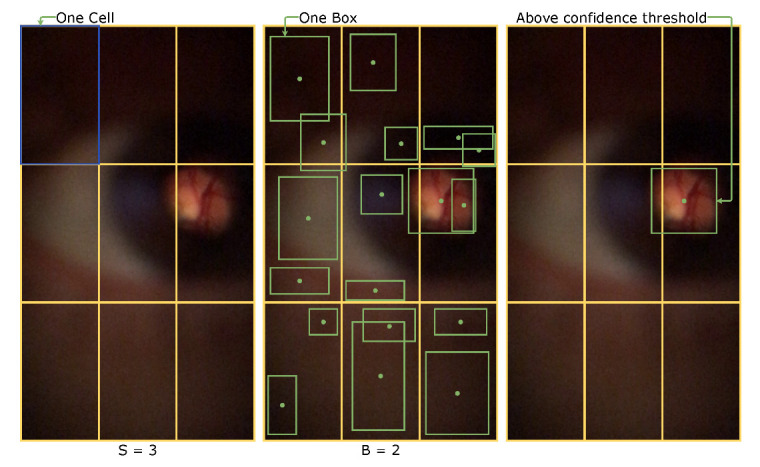
YOLO bounding boxes prediction: Grid division of input (**left**); Bounding box prediction (**middle**); Exclusion of low confidence bounding boxes (**right**).

In particular YOLO (You Only Look Once) [10] is an one-stage architecture used for object detection. It was proposed in 2015 and has had multiple versions that improved its speed and accuracy. This network divides the image into an S×S grid. In Figure 2 (left) this division is represented with S equal to 3. Every one of these cells will have B predicted bounding box; similarly in Figure 2 (middle) each cell has two bounding box-B is equal to 2. The limit of each bounding box can cross the cell’s border as long as its center stays inside the cell. After predicting all the bounding boxes, a threshold excludes poorly marked findings. It makes that only predictions with a high confidence score are not suppressed. Then it is applied a non-max suspension to remove duplicated boxes. Figure 2 (right) shows what should be the final result.

All the encoded bounding boxes will have five values in their output: one for confidence score and four numbers to define the bounding box’s limits. For each cell, the result will also contain a C number of values that gives the detected object the probability of belonging to each particular class.

Summarily, the output will be a tensor of S×S×((*x*, *y*, *h*, *w*, *pc*)×B+C), where:

- S×S is the number of columns/rows which the image is divided;
- *x*, *y* are the center coordinates of the bounding box;
- *h*, *w* are the height and width, respectively, of the bounding box. These values fluctuate from 0 to 1 as a ratio of the image height or width;
- *pc* is the confidence score, the probability of a bounding box contains an object;
- *B* is the number of bounding box that each cell contains;
- *C* is the number of classes that the model is trained to detect. Will return the probability of each cell contains an object.

### 2.2. Mosaicing

Mosaicing is a technique used to merge multiple images into a single one [11]. This method is typically divided into two steps, image registration and image blending. Although there is a vast literature about both steps, image registration is considered the fundamental step since it is impossible to proceed to the second step without image alignment. Due to this particularity of mosaicing, a greater focus will be applied to image registration description, and image blending will be only briefly described.

In the image registration step from two images, the algorithm transforms one to match similarities between them in the same referential. Before the emergence of convolutional neural networks in 2012, image registration was done with traditional feature-based approaches. Typically consisted of finding key points (similarities) between near consecutive frames and then describing each of them using, for example, SIFT or other methods. Independently of the approach, after detecting and characterizing each point, matching both images was done to obtain the correct correspondence of points. Based on the correspondences, a warp is performed in one of the images to have equal proportions of objects and be placed in the same coordinate system. It is relevant to extract quality key points from the images to produce helpful and meaningful mosaicing, i.e., find enough correspondence points in the input images to merge.

In particular GLAMpoints [7] is a keypoint detector and uses root-SIFT to do the description of the interest points. An advantage of this network is that it uses unsupervised learning. Next, a transform is applied for each dataset image, obtaining two images and a matrix of homography between them as ground truth. The authors point out as a problem of other detectors the enormous density of key points increasing the false matches and claim good results compared to others. Unfortunately, the lack of a public dataset for results comparison and the lack of a metric to know if the output of the net is a good result are points that must be improved from this paper.

After image registration, the overlap of the transformed images needs further attention. Due to different colors intensities or irregularities in the transition between images, the borders of the images meet will need to be smoothed. This step is known as image blending or stitching and can be divided into four classes: feathering-based, pyramid-based, gradient-base, and optimal seam-based blending, as presented in [12,13].

## 3. Materials and Methods

**Figure 3:**
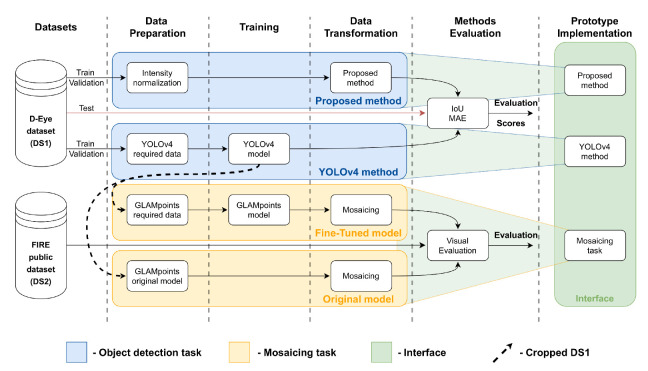
Methodology pipeline.

Figure 3 presents an overview of how this work was implemented and how this section was divided into subsections to be more easily explained. As depicted in Figure 3, two different methods were developed for the retinal detection task applied to D-Eye-based images. The first method is based on a classical image processing approach, and during this paper, it will be named “Proposed method”. The second method for object detection was implemented using a state-of-the-art network, the YOLOv4.

For the mosaicing task, we developed two approaches as well. In the first approach (named “Fine-Tuned model”) were made a fine-tune of the original GLAMpoint model [7], trained and made available. It was used only the retinal area of each frame from the D-Eye dataset (DS1) to perform this task. In the second approach (named “Original model”), the original GLAMpoints model was used to warp cropped images from the D-Eye dataset and to perform the mosaicing task from the videos.

### 3.1. Datasets

Two retinal datasets were used in the development of this work. One is private, the D-Eye dataset [6], and the other is public, the FIRE dataset [5].

The D-Eye dataset will be named DS1 throughout this document, containing 26 low-resolution retinal videos of the optic disc with a resolution of 1920 × 1080 pixels, and it is annotated with the localization of the visible retina area. The dataset is divided into folders as stated in Table 1. The number of images that constitutes the DS1 is not proportional to the number of videos because some present lack quality, in terms of focus or lack of illumination, or present lack of information (for example, the patient’s eye was closed. They weren’t considered suitable to apply in the neural network for those reasons. The DS1 was used in the object detection and mosaicing algorithms analyzed in this work for training and evaluation.

The FIRE dataset [5] will be named DS2 throughout this document, which has 134 image pairs with 45º of FOV and a resolution of 2912 × 2912 pixels. These pairs of images are divided into three categories taking into account their anatomic changes and their level of overlapping. The S category has 71 image pairs with increased overlap and small anatomical changes between images. The P category comprises 49 pairs, and the images have reduced overlap compared to the S category and keep small changes between images. The last 14 pairs are in the A category. They have increased overlap and significant anatomical changes between images. Table 2 summarizes the previously mentioned category separation.

The DS2 was applied to the mosaicing algorithm for the visual evaluation of the GLAMpoints models. Since this model was not used for any NN model training, the folder division into train, validation, and test was unnecessary for this dataset.

### 3.2. Data Preparation

The data preparation step is where all the required data will be organized and prepared to implement the methods under analysis. The two techniques implemented for retinal localization detection are pretty different from each other, resulting in additional requirements in terms of data preparation. For the object detection task, it was only used the dataset DS1.

#### 3.2.1. Proposed Method

Due to the non-constant color intensity of the DS1 images, the data preparation for the Proposed method is to normalize the intensity of all the used images. This process aims to prepare and transform the data for a better overall result. Based on the [12] approach, this problem’s correction was made by performing the normalization of all the dataset’s image average intensity. The normalization of each image is described with Equation (1). To perform the normalization task, firstly, is calculated the mean intensity of each analyzed image, *I*${}_{Xmean}$. Then, the lowest mean of pixel intensities is found, *I*${}_{min}$. That value is divided by each image’s mean pixel intensity, obtaining a list of ratio values. Each of the ratio values is then multiplied by its correspondent image (*Fig*${}_{X}$ in the equation), obtaining a normalized image (*Fig*${}_{Xnorm}$).

(1) $$Fig_{Xnorm}=\frac{I_{min}}{I_{Xmean}}Fig_{X}$$

This process changes the intensity of all images to the same value in every frame. This task can take a while to complete if many pictures are being processed, nevertheless bringing better results.

#### 3.2.2. Yolo V4 Network

Unlike the classical approach from the Proposed method, neural networks are robust to image intensity changes. If the model is well trained, the expected results will be typically better than those based on image transformations. For a custom-trained model, specific data preparation is needed and framework-dependent.

To train the YOLO v4 model correctly, the original configuration (.cfg file), provided by the author, was updated. Firstly, the batch parameter was defined as 64 and the subdivisions as 16. Next, width and height values were determined with 416 pixels, a standard value. The remaining changes were done based on the number of object classes necessary to detect. In this case, it is intended to find only one object, the retina, so the number of classes was defined as 1. In the three convolutional layers section, the max_batches, steps, and filters values were changed before the YOLO layers. The max_batches value is defined by multiplying the number of classes by 2000, but this value must never be less than 6000. This way, it was described as 6000. The steps parameter has two values. The lower must be equivalent to 80% of max_batches, and the higher must be equal to 90% of max_batches. So, they were defined as 4800 and 5400. The filters value is obtained by multiplying the number of classes plus five by three. The value of filters for one class is 18.

#### 3.2.3. Mosaicing

**Figure 4:**
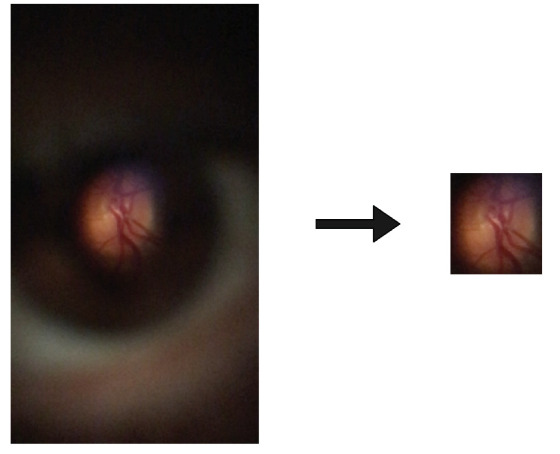
Example of how the D-Eye images were cropped. At **left** is presented a D-Eye image and at **right** is presented the result after data preparation.

The mosaicing task will use the two datasets, DS1 and DS2, to implement the methodology described in this chapter. It was used only the retinal area of the DS1 images to fine-tune the GLAMpoints original model. Next, the YOLOv4 network was used to identify and crop the retinal regions from all the pictures of DS1. Finally, the images were cropped, and padding was added to all the images that have the same spatial size of 400 × 400 pixels, keeping the retina’s original size and scale (corresponds to the Cropped DS1 in Figure 3). Figure 4 presents an example of which information was considered important from D-Eye images to perform the mosaicing task.

The dataset DS2 was resized to 15% of its original size, similarly to the approach presented in the Truong et al. [7] for increased computational performance. As a result, all the images were resized to the size of 436 × 436 pixels. This dataset was only used for the visual evaluation of the mosaicing models.

### 3.3. Training

**Figure 5:**
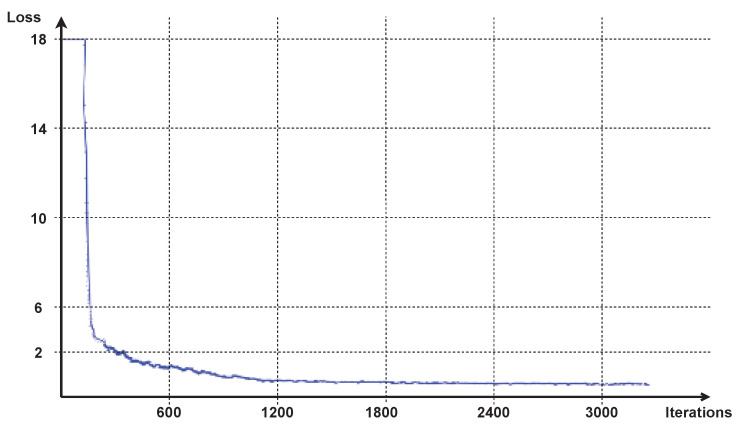
Chart of loss from YOLOv4 model training.

In this work, two models were trained, the YOLO v4 network for the object detection task and the GLAMpoints network for keypoint detection to apply in a mosaicing technique. Both models were trained using Google Colab. The image’s datasets, ground-truths, and configuration files were all uploaded to Google Drive. Then was installed on Colab, the OpenCV, CUDA, CUDNN, and Darknet. Finally, the YOLO v4 model was trained over the COCO dataset’s pre-trained weights [14]. After the training in Google Colab, the models were automatically saved to Google Drive, and the following chart was obtained, which depicts the average loss versus the number of iterations, Figure 5. The training of the model was stopped after 3300 iterations.

In the mosaicing task, the original model provided by Truong et al. was fine-tuned with the cropped retinal areas of the DS1. First, the YOLO v4 previously trained model was used to get the Bounding Boxes (BB) coordinates that encapsulate the retina. Then, those coordinates were used to crop each of the DS1 images, creating a new dataset from DS1 only with the low-resolution retinal areas (Cropped DS1 in Figure 3).

The network was trained multiple times, and for the training results comparison, it was used the tool WandB [15], which allowed the tracking of the training data results. As D-Eye images have fewer frame-to-frame transformations, it won’t be necessary to perform high demanding training (with high transformation values). This way, several training sessions were carried out with controlled data augmentation, activating and deactivating the parameters presented in Table 3. Other parameters were kept constant, for example, the image size of 256 × 256 pixels, the learning rate of 0.001, and the number of epochs as 14.

**Figure 6:**
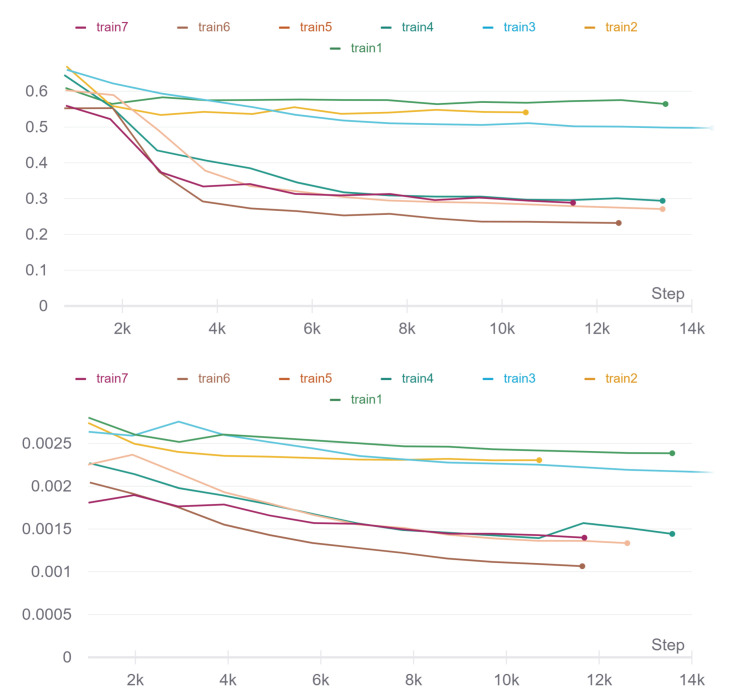
Train loss graphic (**top**) and validation loss graphic (**bottom**).

With the reduction in data augmentation parameters, there are fewer transformations in the images, and it will be easier to perform the homography in the training process. It is reflected in the loss graphs shown in Figure 6, where training with fewer transformations presents a greater convergence than those using more transformations.

### 3.4. Data Transformation

This stage of the methodology section is reserved for explaining the additional transformations of the data before the evaluation step after this step is expected that the object detection task outputs the retinal location and that the mosaicing task to have an image.

#### 3.4.1. Proposed Method

In the data transformation step of the Proposed method, the objective is to extract the location of the retina from the normalized images of the DS1. To extract the retina location and since the retina has a circular shape in the images, it was decided to apply the Circle Hough Transform (CHT) [16] algorithm to the images. The CHT finds centers and radius circles from edged (contour) images. Therefore, to find the location of the retina with the CHT is required to transform the DS1 photos into contour images with highlighted retinal areas.

**Figure 7:**
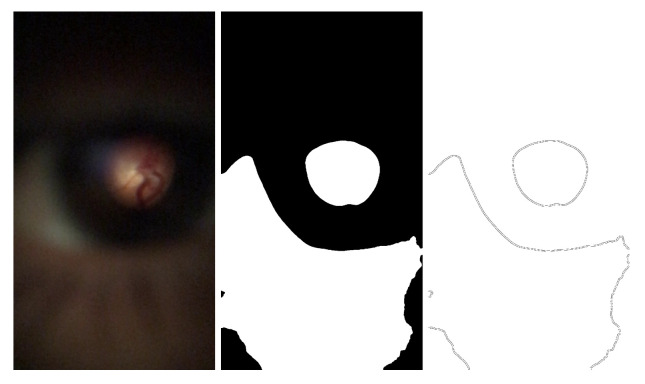
Example of an image where the Proposed method was applied (**left**); Result of the Otsu’s threshold (**middle**); Contour image after adaptive threshold (**right**).

In the first step, the extraction of the red channel in the RGB color space was performed after verifying that important information was kept in the color channel. The image is then blurred, as a second step, before applying a threshold. This blurring procedure resulted in images with smoother edges (that revealed to obtain better results after applying CHT in later phases). The Otsu method [17] was chosen as the threshold method in the third step since it is an adaptive threshold. To be adaptive means that a different threshold value will be automatically calculated depending on the image profile. The result of this step is a binary image, as presented in the example in Figure 7 (middle). Afterward, in step four, the contours are extracted from the binary image, creating a new binary image. Such a generated image is binary and has a white background and black lines. The lines in the image should be marking the retina region with a well-defined circle, and the other shapes should be discarded, similarly to Figure 7 (right).

As mentioned, since the retina is circular, it is possible to apply a CHT and proceed with the location’s extraction. CHT outputs the coordinates of the center of the detected circle and its radius. From these values, the square enclosing the circle is obtained.

#### 3.4.2. Mosaicing

Mosaicing is a technique that can be divided into two main steps: image registration, where image’s keypoints are found and images are warped, and image blending, where image borders are smoothed.

The first step of this mosaicing algorithm is to normalize the intensities of all the images. This step is done for a smoother transaction between image borders in the final result. After this, the image registration step is implemented, which uses the network GLAMpoints [7] to obtain keypoint correspondences between two images. In this network, the keypoints description is done using SIFT. These two images will be identified as “base image” used as reference and “following image” that will be warped in correspondence to the similarities between images. As a result, the two images with similarities will match in the same referential, and the image registration step is done.

The next step is image blending. In this stage was implemented the featuring-based method as presented by Melo et al. [12], which creates two weighted masks to achieve a smoother look to the transition between images. Firstly, it is found the intersection between the images and, for each pixel inside of the overlapping region, are applied the Equations (2) and (3).

(2) $$w1\left(x,y\right)=\left\{\begin{array}{cc} 0 & \mathrm{if}\;m1(x,y)=0\\ \frac{d2}{d1+d2} & \mathrm{if}\;m1(x,y)=1⋀R(x,y)=1\\ 1 & \mathrm{otherwise} \end{array}\right.$$

(3) $$w2\left(x,y\right)=\left\{\begin{array}{cc} 0 & \mathrm{if}\;m2(x,y)=0\\ \frac{d1}{d1+d2} & \mathrm{if}\;m2(x,y)=1⋀R(x,y)=1\\ 1 & \mathrm{otherwise} \end{array}\right.$$

(4) I(x,y)=w1(x,y)∗I1(x,y)+w2(x,y)∗I2(x,y)

These equations describe the Euclidean distance between each pixel and the closest pixel of that image outside the overlapping region. After applying this to all the intersection pixels is applied the Equation (4), which multiplies the weighted masks to their correspondent warped image to obtain the final image with smooth transitions, and is named as the “result image”.

**Figure 8:**
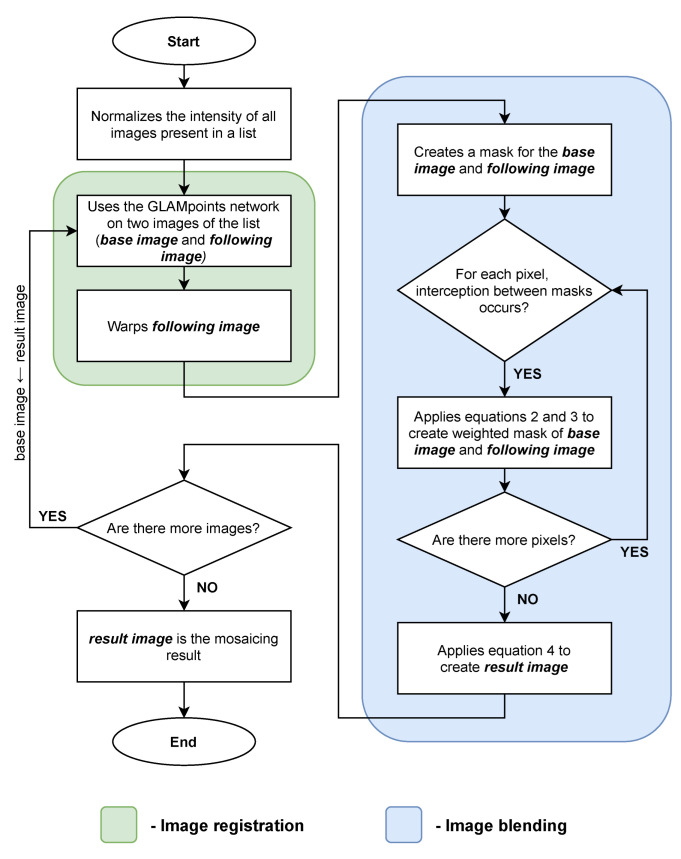
Image mosaicing flowchart.

Figure 8 presents the overall scheme that has been described in the previous paragraphs to implement the mosaicking technique.

### 3.5. Evaluation

Metrics are a way to measure the performance of a process. It is difficult to affirm that a model surpasses others since quantitative appraisal does not exist without the evaluation through metrics. This subsection presents the metrics used to evaluate the retinal detection methods. In mosaicing, a quantitative assessment was not implemented. Only a visual evaluation was performed.

The D-Eye dataset includes BB location ground truth, and since the Proposed method and YOLO v4 return BBs, it is possible to present the comparative results of the methods. Two metrics were used in this work, the Mean Absolute Error (MAE) [18] and Intersection over Union (IoU) [19].

The Mean Absolute Error is a simple calculation. Firstly, from the ground-truth value $y_{i}$ is subtracted the predicted value $x_{i}$, resulting in a predicted error. Then, this predicted error is calculated its magnitude, turning it into an absolute value. This procedure is done four times (*n* = 4) per image, one for each corner of the bounding box’s coordinates. Finally, the mean value between the four absolute values is calculated, resulting in that image’s MAE value, as presented in Equation (5). MAE results will be proportionately weighted since it is a linear operation. The resulting score of this metric can go from zero to infinite, and lower MAE values represent better model performance. The average value of all the contributions is considered the global MAE performance of the model.

(5) $$\mathrm{MAE}=\frac{\sum_{i=1}^{n}\left|y_{i}-x_{i}\right|}{n}$$

**Figure 9:**
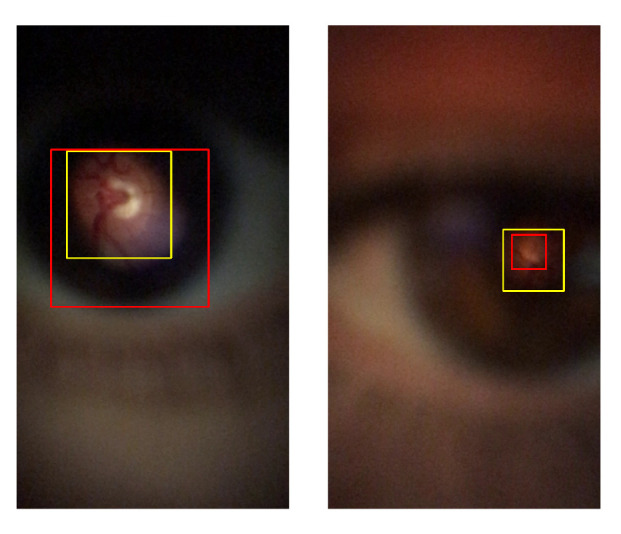
Comparison between MAE and IoU metric results: MAE = 103.0 and IoU = 0.45 (**left**); MAE = 53.0 and IoU = 0.32 (**right**).

The MAE value alone will not be enough to prove that one model is outputting good results. Figure 9 demonstrates one example where the bounding box of the figure at the right is poorly annotated when compared to the figure in the left (yellow square). In Figure 9 at the left, is present all the retina (red square), and in the other case, the retina was cropped. Even though the MAE is lower (wrongly indicates better performance) in Figure 9 (right), the implementation of additional metrics will prevent the wrong analysis of the MAE results.

The other selected metric was IoU. For two finite samples A and B, this metric calculates a ratio of the overlapping area of the samples and the union area of both samples. The IoU value varies between zero and one and indicates the total BB area percentage that overlaps. Higher values will represent better performance. In this equation, A can be considered the ground truth region and B the resulting region from the YOLO v4 method.

Equation (6) calculates the IoU for each image. The average value of the whole set of images is considered the global IoU performance of the model. As can be depicted in Figure 9, this metric attributes the higher value (better performance) to the best image (Figure 9 on the left side).

(6) $$IoU=\frac{A\cap B}{A\cup B}$$

In summary, the two metrics complete each other. While the IoU indicates how good the prediction is, the MAE indicates the prediction deviations.

## 4. Results and Discussion

### 4.1. Retinal Detection

**Figure 10:**
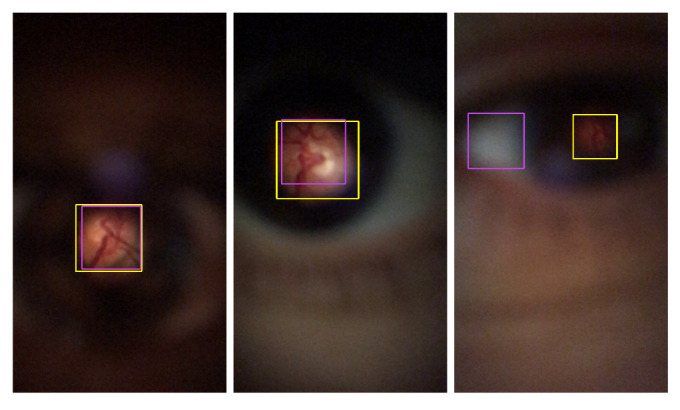
Example of bounding boxes visual results of Zengin et al. [6]. Successful classification (**left**); Acceptable classification (**middle**); Failed classification (**right**).

**Figure 11:**
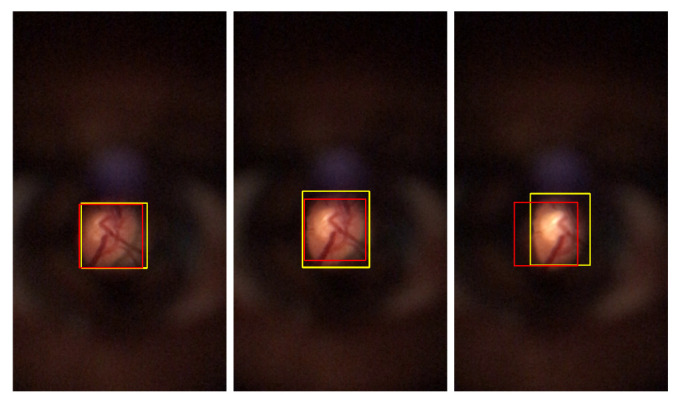
Example of bounding boxes visual results of Proposed method. Successful classification (**left**); Acceptable classification (**middle**); Failed classification (**right**).

**Figure 12:**
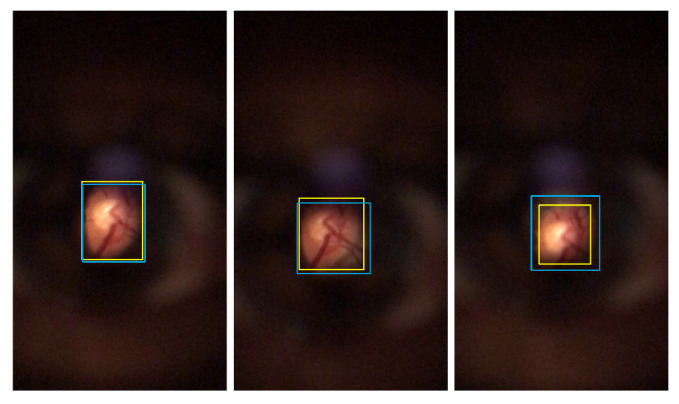
Example of bounding boxes visual results of YOLO v4. Successful classification (**left**); Acceptable classification (**middle**); Failed classification (**right**).

The Proposed method overall results, the trained model of YOLO v4 with DS1, and the Zengin et al. [6] retinal detection task results were directly compared. Based on visual analysis and the IoU values, three ranges were defined for image classification: Successful class is defined for images with IoU greater than 0.8. For IoU results between 0.6 and 0.8 (included), the Acceptable class is assigned. Finally, images with less than or equal to 0.6 are attributed to the Failed class. Figure 10, Figure 11 and Figure 12 present multiple D-Eye examples with bounding boxes marking ROI’s, where the yellow boxes are the ground truth plots. Red boxes are the proposed method results, purple boxes are the Zengin et al. [6] annotations, and blue boxes are the YOLO v4 network outputs. The first column of each of these figures presents the Successful class examples. Acceptable class examples and the Failed class examples are displayed in the last column in the second column.

For all the left, one can observe that most images that express good results from the three comparable methods are quite good, presenting BBs quite similar to the ones from the ground truth (in yellow). Considering the middle images for the Acceptable classification ones can observe a visually poor result for Zengin et al. [6] method, due to the reduced size of the BB when compared to the ground truth, suggesting a higher MAE value and a lower IoU value. For the rightmost column, visually, the most acceptable misclassification would be the one from YOLO v4, where a good portion of the retinal area is detected. For the Proposed method, there’s a shift in the position of the BB, so IoU should be poor (occurs due to the poor results from the Circle Hough Transform, a consequence of the pre-processing steps in the Proposed method). The result of Figure 10, is an example where the method was unable to detect the retinal area.

Based on those class divisions, Table 4 was compiled, showing the number of images, the average MAE, and average IoU for each classification class, being the best results highlighted in bold. Since the dataset is the same (DS1), a direct comparison of these results can be performed to the ones presented by Zengin et al. [6].

Considering the overall success as the merge of both Successful and Acceptable classes (IoU > 0.6), for Zengin et al. [6] can be observed that 84.3% were successfully classified, although only being 44.7% from Successful class. With a slightly lower performance, there are the results associated with the Proposed method, where 40.9% of the images are classified as Successful and 45.6% as Acceptable. In this case, the cumulative frequency of these two indicators is 86.5%, which slightly improves Zengin et al.’s results [6]. On the other hand, the YOLO v4 network application verified that it presents the best performance since only one image is classified as Failed. As such, 99.9% of the dataset is classified as Successful or Acceptable. It should also be noted that from this 99.9%, around 78.2% are classified as a success, which stands out concerning the other works in comparison, regarding the location of the retinal area. Considering the metrics that assess the quality of the identification of the retinal location, it is observed that for the YOLO v4 network, the best classifications are obtained in terms of MAE, with a value of 11.05 for Successful class (instead of 12.84 for [6] and 13.52 for the Proposed method). For the Acceptable class, the value of 25.73 (instead of 26.69 for [6] and 26.24 for the Proposed method) and for the Failed class the values of 80.27 and 73.99 for [6] and for Proposed method respectively). Similarly, the YOLO v4 network showed an improvement for all classifications considered, with an average IoU value of 0.88 for the Successful class, instead of the 0.85 that the other methods present. For the Acceptable class, the behavior is similar, with 0.75 (instead of 0.73 for [6] and Proposed method) and 0.60 (instead of 0.48) for the Failed class.

Table 5 presents the global results for each method. In this table, it is possible to observe that the Proposed method results are very similar to the results obtained by [6]. However, the proposed method had a substantially better standard deviation value for MAE. Taking into account the YOLO v4 network, it can be seen that it stands out in an improvement, both in the mean values of MAE and IoU and in the standard deviation values, also corroborating the analysis carried out in Table 4.

In summary, both the Proposed method and YOLO v4 brought an improved global result to the work of Zenin et al. [6], and as expected, due to the nature of YOLO v4 as a detector network, it stands out as the best approach to follow.

### 4.2. Mosaicing

Two different approaches were used for the mosaicing procedure, which differs in the GLAMpoints model. The first is a fine-tuned model applying the DS1 cropped dataset, and the second was used the original model trained by Truong et al. [7].

**Figure 13:**
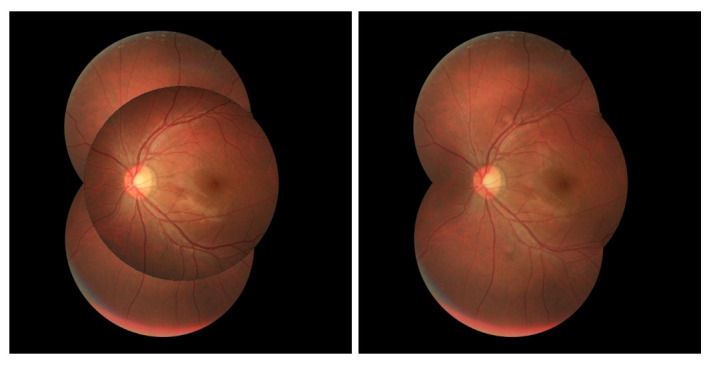
Mosaicing result obtained with the original model of GLAMpoints applied to three images of DS2: Image registration result (**left**); Image blending result (**right**).

As stated before, the mosaicing procedure can be divided into image registration and image blending tasks. Figure 13 (left) presents the results for an image registration step between three DS2 [5] high-quality images. This image was obtained applying the image registration implementation to the original GLAMpoints model. The image blending step was applied to eliminate the transitions between optic discs, resulting in Figure 13 (right). The latter presents a smoother transition when aggregating all the contributions.

**Figure 14:**
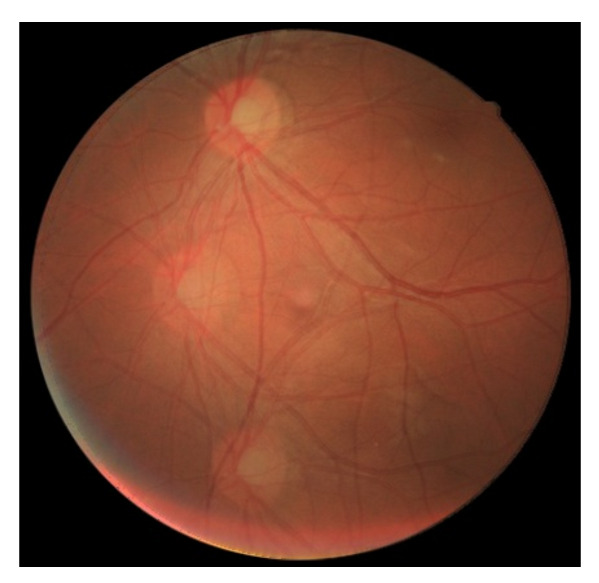
Mosaicing result obtained with the fine-tuned model of GLAMpoints applied to the same three images of DS2.

Figure 14 presents the mosaicing result executed with the fine-tuned model, applied to the same three images of the DS2 dataset previously refereed. It is possible to observe that the fine-tuned model made the results quite worse than the result of Figure 13 (right). Using the cropped DS1 dataset, the fine-tuned model made adjustments in the model’s weights to detect key points in images with lower quality, although losing its ability to correctly detect key points in higher quality images like the ones from DS2.

**Figure 15:**
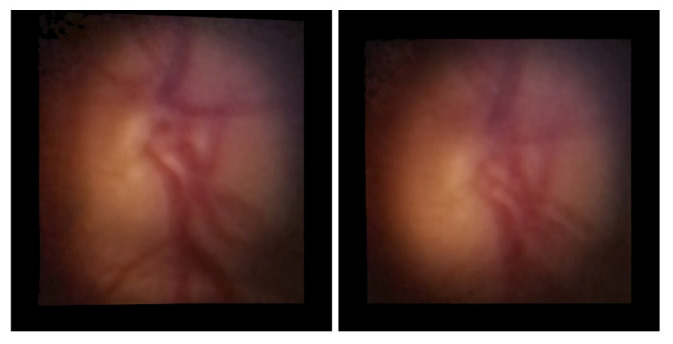
Comparison between mosaicing of the DS1 cropped images: Using the original model (**left**) and using the fine-tuned model (**right**).

Figure 15 presents the comparison between mosaicing using the original and the fine-tuned models in DS1 cropped images. As can be observed, the original model got better visual results than the fine-tuned. The original GLAMpoints model could easily find key points in DS2 photos. With the fine-tuning of the original model, it was intended to adapt the weights to find key points in cropped DS1. As can be observed, the set of training images provided may not have been sufficient to adjust the model to find relevant keypoint in D-Eye images, resulting in worse results.

## 5. Conclusions

The equipment to capture retinal fundus images are expensive, and due to its size, they possess a lack of portability. The development of technology brought new alternatives for screening that can be attached to smartphones, being one example the D-Eye device. Compared to specialized equipment, the D-Eye presents lower quality in the captured retinal video yet is enough to perform a medical pre-screening. The idea of using D-EYE or similar technologies is not to substitute the medical diagnosis in any way. This pre-screening technology will allow that a person can be checked in situ. Often, the person under analysis didn’t yet understand or notice its lack of vision due to glaucoma. This pre-screening will inform if there is the need to further extend their eye examinations in a specialized center, with professional assistance. This work contributes to implementing methods to more effectively use the images captured by D-Eye, extracting useful information from the low-quality D-Eye retinal videos and then merging multiple frames into a single one. This chapter aims to summarize the contributions made with this work and address some points that require attention and would be interesting to be analyzed and developed in the future.

The first contribution was to evaluate object detection methods to extract D-Eye relevant information, i.e., retinal location. It is an essential task since a large area of D-Eye images has no relevant information, being the retinal (relevant information) only a tiny portion of the picture. Another contribution was to apply the mosaicing technique to the cropped D-Eye images, i.e., merge multiple retinal photos to create a single and more informative picture. This way, it would be possible to obtain a single summary image from the D-Eye videos.

In the Object detection contribution, two different methods were implemented with success for retinal region detection in D-Eye images. The first was purely developed based on a classical programming approach using CHT. The second was done training a model of YOLO v4 with the D-Eye dataset [6]. When comparing the performance of the two implemented methods with the object detection implemented by Zengin et al. [6], it is noticeable an improvement in the region proposals. In that work, Zengin et al. presented results with an IoU of 0.75 and a standard deviation of 0.14. The Proposed method showed a slight improvement reducing the standard deviation to 0.13. The trained YOLO v4 model obtained better results with an IoU of 0.85 and a standard deviation of 0.07.

In the mosaicing, the process was divided into two stages: image registration and image blending. First, in image registration, key points were found using the GLAMpoints network [7] and then described with SIFT descriptor. Then, after the warp of the images to have the same coordinate system, image blending was applied to smooth the transition between images, using a feature-based method. Due to a lack of progress in the results of the fine-tuned model trained with the cropped retinal area images of the D-Eye dataset, mosaicing was considered a success. However, it needs model improvement for D-Eye images since the one used was obtained from the Truong et al. [7].

## Figures and Tables

**Table 1:** D-Eye dataset division in train, validation, and test sets—adapted from [6].

	Resolution (Pixels)	Train	Validation	Test
**DS1**	1920 × 1080	18 videos; 3881 images	3 videos; 776 images	5 videos; 1375 images

**Table 2:** FIRE public dataset division in categories.

	Resolution (Pixels)	S Category	P Category	A Category
**DS2**	2912 × 2912	71 pairs	49 pairs	14 pairs

**Table 3:** Multiple parameter variations in GLAMpoints network training.

Name	Use Green Channel	Use Rotation	Use Scaling	Use Perspective	Use Shearing
Train 1	no	yes	yes	yes	yes
Train 2	no	yes	yes	yes	yes
Train 3	no	no	yes	yes	yes
Train 4	no	no	no	yes	yes
Train 5	no	no	no	no	yes
Train 6	no	no	yes	yes	no
Train 7	yes	no	no	yes	yes

**Table 4:** Comparison between methods, for Successful, Acceptable and Failed class results.

	SUCCESSFUL (IoU > 0.8)				ACCEPTABLE (0.6 <IoU ≤ 0.8)				FAILED (IoU ≤ 0.6)			
	Frequency		MAE	IoU	Frequency		MAE	IoU	Frequency		MAE	IoU
	Absolute	Relative			Absolute	Relative			Absolute	Relative		
Zengin et al. [6]	615	44.73%	12.84	0.85	544	39.56%	26.69	0.73	216	15.71%	80.27	0.48
Proposed method	562	40.87%	13.52	0.85	**627**	**45.60%**	26.24	0.73	186	13.53%	73.99	0.48
YOLO v4	**1075**	**78.18%**	**11.05**	**0.88**	299	21.75%	**25.73**	**0.75**	**1**	**0.07%**	**40.25**	**0.60**

**Table 5:** Overall results (average and standard deviation) for methods to be compared.

		Mean	Standard Deviation
Zengin et al. [6]	MAE	28.91	47.04
	IoU	0.75	0.14
Proposed method	MAE	27.5	29.29
	IoU	0.75	0.13
YOLO v4	MAE	**14.26**	**7.92**
	IoU	**0.85**	**0.07**

## Data Availability

Not applicable.

## Abbreviations

The following abbreviations are used in this manuscript:

BB	Bounding Box
CHT	Circle Hough Transform
FOV	Field of View
IoU	Intersection over Union
MAE	Mean Absolute Error
RoI	Region of Interest
SIFT	Scale-invariant feature transform
YOLO	You Only Look Once

## References

[1] Russo A., Morescalchi F., Costagliola C., Delcassi L., Semeraro F. (2015). A novel device to exploit the smartphone camera for fundus photography. J. Ophthalmol..

[2] Maamari R.N., Keenan J.D., Fletcher D.A., Margolis T.P. (2014). A mobile phone-based retinal camera for portable wide field imaging. Br. J. Ophthalmol..

[3] Inview®. https://www.volk.com/collections/diagnostic-imaging/products/inview-for-iphone-6-6s.html.

[4] Wu A.R., Fouzdar-Jain S., Suh D.W. (2018). Comparison study of funduscopic examination using a smartphone-based digital ophthalmoscope and the direct ophthalmoscope. J. Pediatr. Ophthalmol. Strabismus.

[5] Hernandez-Matas C., Zabulis X., Triantafyllou A., Anyfanti P., Douma S., Argyros A.A. (2017). FIRE: Fundus image registration dataset. J. Model. Ophthalmol..

[6] Zengin H., Camara J., Coelho P., Rodrigues J.M., Cunha A. (2020). Low-Resolution Retinal Image Vessel Segmentation. Proceedings of the International Conference on Human-Computer Interaction.

[7] Truong P., Apostolopoulos S., Mosinska A., Stucky S., Ciller C., Zanet S.D. GLAMpoints: Greedily Learned Accurate Match points. Proceedings of the IEEE International Conference on Computer Vision.

[8] Liu L., Ouyang W., Wang X., Fieguth P., Chen J., Liu X., Pietikäinen M. (2020). Deep learning for generic object detection: A survey. Int. J. Comput. Vis..

[9] Jiao L., Zhang F., Liu F., Yang S., Li L., Feng Z., Qu R. (2019). A survey of deep learning-based object detection. IEEE Access.

[10] Redmon J., Divvala S., Girshick R., Farhadi A. You only look once: Unified, real-time object detection. Proceedings of the IEEE Conference on Computer Vision and Pattern Recognition.

[11] Patton N., Aslam T.M., MacGillivray T., Deary I.J., Dhillon B., Eikelboom R.H., Yogesan K., Constable I.J. (2006). Retinal image analysis: Concepts, applications and potential. Prog. Retin. Eye Res..

[12] Melo T., Mendonça A.M., Campilho A. (2018). Creation of Retinal Mosaics for Diabetic Retinopathy Screening: A Comparative Study. Proceedings of the International Conference Image Analysis and Recognition, Póvoa de Varzim.

[13] Ghosh D., Kaabouch N. (2016). A survey on image mosaicing techniques. J. Vis. Commun. Image Represent..

[14] Lin T.Y., Maire M., Belongie S., Hays J., Perona P., Ramanan D., Dollár P., Zitnick C.L. (2014). Microsoft Coco: Common objects in context. Proceedings of the European conference on Computer Vision.

[15] WandB. http://www.wandb.ai.

[16] Kimme C., Ballard D., Sklansky J. (1975). Finding circles by an array of accumulators. Commun. ACM.

[17] Otsu N. (1979). A threshold selection method from gray-level histograms. IEEE Trans. Syst. Man. Cybern..

[18] Willmott C.J., Matsuura K. (2005). Advantages of the mean absolute error (MAE) over the root mean square error (RMSE) in assessing average model performance. Clim. Res..

[19] Zhou D., Fang J., Song X., Guan C., Yin J., Dai Y., Yang R. IoU loss for 2d/3d object detection. Proceedings of the 2019 International Conference on 3D Vision (3DV).

